# Retraction: Design and development of highly sensitive PEDOT-PSS/AuNP hybrid nanocomposite-based sensor towards room temperature detection of greenhouse methane gas at ppb level

**DOI:** 10.1039/d5ra90001h

**Published:** 2025-01-09

**Authors:** Syed Khasim, Apsar Pasha, Nacer Badi, Adnen Ltaief, S. A. Al-Ghamdi, Chellasamy Panneerselvam

**Affiliations:** a Department of Physics, Faculty of Science, University of Tabuk Tabuk-71491 Kingdom of Saudi Arabia syed.pes@gmail.com; b Renewable Energy Laboratory, Nanotechnology Research Unit, University of Tabuk Tabuk-71491 Kingdom of Saudi Arabia; c Department of Physics, Ghousia College of Engineering Ramanagaram-562159 Karnataka India; d Department of Biology, Faculty of Science, University of Tabuk Tabuk-71491 Kingdom of Saudi Arabia

## Abstract

Retraction of ‘Design and development of highly sensitive PEDOT-PSS/AuNP hybrid nanocomposite-based sensor towards room temperature detection of greenhouse methane gas at ppb level’ by Syed Khasim *et al.*, *RSC Adv.*, 2021, **11**, 15017–15029, https://doi.org/10.1039/D1RA00994J.

The Royal Society of Chemistry hereby wholly retracts this *RSC Advances* article due to concerns with the reliability of the data.

The SEM image in Fig. 3b is identical to Fig. 3a of ref. 1, with no authors in common. The authors say that the image was provided by a 3rd party but are unable to provide the raw data. The authors have provided a new SEM image for consideration, but after review by an independent expert, it was determined that the new image is not suitable as it does not provide enough clarity on the particles’ shape and size.

The FTIR spectra in Fig. 4a has unusual features, and the raw data provided does not match with the published data.

Given the significance of these concerns, the findings presented in this paper are no longer reliable.

This retraction supersedes the information provided in the Expression of Concern related to this article.

The authors were informed about the retraction of the article, but have not responded.

Retraction endorsed by Laura Fisher, Executive Editor, *RSC Advances*

Date: 19th December 2024
